# Norm Values and Psychometric Properties of the 24-Item Demoralization Scale (DS-I) in a Representative Sample of the German General Population

**DOI:** 10.3389/fpsyg.2021.681977

**Published:** 2021-06-14

**Authors:** Leonhard Quintero Garzón, Andreas Hinz, Susan Koranyi, Anja Mehnert-Theuerkauf

**Affiliations:** Department of Medical Psychology and Medical Sociology, University Medical Center Leipzig, Leipzig, Germany

**Keywords:** demoralization, general population, validation, normative study, existential distress

## Abstract

**Purpose:** The Demoralization scale (DS-I) is a validated and frequently used instrument to assess existential distress in patients with cancer and other severe medical illness. The purpose of this study was to provide normative values derived from a representative German general population sample and to analyze the correlational structure of the DS-I.

**Methods:** A representative sample of the adult German general population completed the DS-I (24 Items), the Emotion Thermometers (ET) measuring distress, anxiety, depression, anger, need for help, and the Functional Assessment of Chronic Illness Therapy Fatigue Scale (FACIT-fatigue).

**Results:** The sample consists of *N* = 2,407 adults (mean age = 49.8; range = 18–94 years), 55.7% women). The percentages of participants above the DS-I cutoff (≥30) was 13.5%. The mean scores of the DS-I dimensions were as follows: (1) loss of meaning and purpose: *M* = 2.78 *SD* = 4.49; (2) disheartenment: *M* = 3.19 *SD* = 4.03; (3) dysphoria *M* = 4.51 *SD* = 3.20; (4) sense of failure: *M* = 6.24 *SD* = 3.40; and for the DS-I total score: *M* = 16.72 *SD* = 12.74. Women reported significantly higher levels of demoralization than men, with effect sizes between *d* = 0.09 (Loss of Meaning) and *d* = 0.21 (Dysphoria). Age was not associated with demoralization in our sample. DS-I reliability was excellent (α = 0.94) and DS-I subscales were interrelated (*r* between 0.31 and 0.87) and significantly correlated with ET, especially depression, anxiety, and need for help and fatigue (*r* between 0.14 and 0.69).

**Conclusions:** In order to use the DS-I as a screening tool in clinical practice and research the normative values are essential for comparing the symptom burden of groups of patients within the health care system to the general population. Age and sex differences between groups of patients can be accounted for using the presented normative scores of the DS-I.

## Introduction

Demoralization describes a syndrome of existential distress and despair. It encompasses feelings of entrapment and alienation, states of helplessness and hopelessness with loss of meaning and purpose in life (Kissane et al., 2001). Demoralization is represented on a spectrum from disheartenment to despondency and despair to the demoralization syndrome (Figueiredo, 2013; Grassi and Nanni, 2016).

Demoralization has been frequently studied in the context of severe physical illness (Robinson et al., 2015). Due to its relatively high prevalence of 13–33% in patients with a progressive disease such as cancer (Robinson et al., 2015) and its profound impact on a patient's well-being (Vehling and Mehnert, 2014; Vehling et al., 2017) it is gaining increasing attention in clinical practice, particularly in palliative care (Vehling et al., 2013, 2019; An et al., 2018; Bobevski et al., 2018).

Previous studies in cancer patients (Mehnert et al., 2011; Lee et al., 2012), patients with lupus (Katz et al., 2001) or after heart transplant (Grandi et al., 2010) have shown high levels of demoralization in patients who are not married or cohabiting. Full time employment has been shown to be associated with lower demoralization than unemployment in cancer patients (Katz et al., 2001; Lee et al., 2012). Lee et al. (2012) found that low-income earners showed higher demoralization than those with higher income. Regarding age, sex, and education, mixed findings have been reported in the literature (Robinson et al., 2015).

The most widely used self-report questionnaire to assess demoralization symptomatology is the 24-item Demoralization Scale (DS-I) (Kissane et al., 2004). It has been translated and adapted to several languages (Dolbeault et al., 2008; Tang et al., 2011; Rudilla et al., 2016; Grassi et al., 2017) including German (Mehnert et al., 2011). In order to use the DS-I as a screening tool in clinical or research practice, normative values of the general population are essential for assessing and comparing the symptom burden of individuals or specific clinical groups. Normative values for different sex and age groups are highly relevant to better interpret the results obtained with the DS-I in clinical populations. However, empirical data on normative values for the DS-I is limited.

In our present study, we aim (1) to provide normative data on demoralization in the German general population as well as psychometric quality criteria, (2) to examine associations of demoralization with sociodemographic characteristics including age, sex, education, partnership, occupational status, and income, and (3) to examine the extent to which demoralization is related to psychological variables such as distress, depression, anxiety, anger, and need for help as well as fatigue in a representative sample of the German general population.

## Materials and Methods

### Study Participants

Participants of this study were derived from a German household survey that was conducted by a demographic consultation company (USUMA, Berlin) between March and May 2015. The country was separated into 258 areas (sample points) to represent all regions in Germany. These areas were drawn proportionally to the distribution of private households. Once a sample point was selected, streets, houses, households, and household members were chosen randomly. Thus, 4,902 households in 258 sample points representing different regions in Germany were selected. 4,844 households were valid and therefore approached to derive a representative sample of the German general population. 2,513 persons agreed to participate in the study (response rate of 51.9%). Included participants were required to have sufficient German language skills and an age of at least 14 years. Study assistants visited the participants, provided information about the study and handed out the self-report questionnaires. All participants, or caretakers of those that were minors, provided written informed consent to partake in the study. For the analyses participants <18 years (*N* = 76) were excluded. In a second step participants with more than one item missing for one of the subscales of the DS-I (*N* = 158) were excluded, resulting in a sample size of *N* = 2,407. The study was approved by the Ethics Committee of the Medical Faculty of the University of Leipzig.

### Instruments

#### Demoralization Scale (DS-I)

The German version of the DS-I (Mehnert et al., 2011) is a self-report measure for demoralization symptoms and is derived from the characterization of the demoralization syndrome (Kissane et al., 2001; Clarke, 2011). In order to adapt the original questionnaire (Kissane et al., 2004) for German patients, the scale was translated forward and backward into the German language with support of a native English speaker. The questionnaire contains 24 items and comprises four subscales: (1) loss of meaning and purpose (α = 0.88), (2) disheartenment (α = 0.88), (3) dysphoria (α = 0.80), and (4) sense of failure (α = 0.76) and exhibits a good overall internal consistency (α = 0.84). Items are rated on a 5-point Likert scale ranging from 0 (never) to 4 (all the time). A total score for demoralization is calculated by summarizing the single subscale scores. Higher scores indicate higher levels of demoralization. In studies with cancer patients scores ≥30 (Kissane et al., 2004) were used as indicator for clinically relevant moderate demoralization and scores ≥36 for high demoralization (Vehling et al., 2013; Vehling and Mehnert, 2014). The questionnaire was validated (Mehnert et al., 2011) with a sample of advanced cancer patients (*n* = 516).

#### Emotion Thermometers (ET)

Distress, depression, anxiety, anger, and need for help were assessed with the Emotion Thermometers (Mitchell et al., 2010, 2012). It is a tool of five visual-analog self-report scales to measure distress, depression, anxiety, anger, and need for help on 11-point (0–10) scales which are visualized as thermometers. Higher numbers indicate higher degrees of distress, depression, anxiety, anger and need for help. It was developed as a supplement of the Distress Thermometer (National Comprehensive Cancer Network, 2003; Mehnert et al., 2006), which is widely used as a screening tool in clinical oncological practice (Mitchell, 2007; Donovan et al., 2014; Mehnert et al., 2018).

#### Functional Assessment of Chronic Illness Therapy Fatigue Scale (FACIT-Fatigue)

The FACIT-F questionnaire (Yellen et al., 1997; Cella et al., 2002) was included to test the associations between fatigue and demoralization. The FACIT-F is a 13-item questionnaire assessing self-reported fatigue and its impact on daily activities and function. It uses a five-point Likert-type scale (0 = not at all; 1 = a little bit; 2 = somewhat; 3 = quite a bit; and 4 = very much). The scale range is 0–52, with 0 being the worst possible score and 52 being the best possible score indicating no fatigue. Cronbach's α is 0.90 in the German general population (Montan et al., 2018).

### Statistical Analyses

To describe sample characteristics we calculated distribution in age groups, frequencies of relationship status, education, occupational status and income for both sexes and overall. Sample characteristics are shown in Table 1.

**Table 1:** Sociodemographic sample characteristics (*N* = 2407).

	**Men**		**Women**		**Total sample**	
	**(*****N*** **=** **1,066)**		**(*****N*** **=** **1,341)**		**(*****N*** **=2,407)**	
	***N***	**(%)**	***N***	**(%)**	***N***	**(%)**
**Age (years)**						
Mean	49.63		49.95		49.80	
(*SD*)	(17.22)		(17.49)		(17.37)	
**Age group**						
<30 years	174	(16.3)	209	(15.6)	383	(15.9)
30–39 years	163	(15.3)	213	(15.9)	376	(15.6)
40–49 years	182	(17.1)	238	(17.7)	420	(17.4)
50–59 years	211	(19.8)	249	(18.6)	460	(19.1)
60–69 years	182	(17.1)	217	(16.2)	399	(16.6)
≥70 years	154	(14.4)	215	(16.0)	369	(15.3)
**Partnership**						
Living with partner	661	(62.0)	696	(51.9)	1,357	(56.4)
Living without partner	405	(38.0)	645	(48.1)	1,050	(43.6)
**Education**						
<10 years	386	(36.5)	451	(33.6)	837	(34.8)
10–11 years	407	(38.1)	582	(43.4)	989	(41.1)
≥12 years	273	(25.4)	308	(23.0)	581	(24.1)
**Occupational status**						
Working full time/part time	633	(59.4)	650	(48.5)	1,283	(53.3)
Unemployed	67	(6.3)	67	(5.0)	134	(5.6)
Retired	292	(27.4)	383	(28.5)	675	(28.0)
Other	74	(6.9)	241	(18.0)	296	(13.0)
**Income**						
<1,000€	169	(15.9)	581	(43.3)	750	(31.2)
1,000–2,000€	594	(55.7)	640	(47.7)	1,234	(51.3)
>2,000€	303	(28.4)	120	(8.9)	423	(17.6)

*SD, standard deviation*.

Two-factorial ANOVAs were calculated to test for group differences between men and women, as well as age groups regarding the DS-I and its subscales. To test group differences for other sociodemographical variables age group and sex were included as covariables. Effect sizes (Cohen's *d*) were computed using group means with taking into account the dispersion of the group mean values. Pearson correlation coefficients were used to calculate the associations between DS-I scores, FACIT-fatigue, and ET.

## Results

### Sample Characteristics

Sociodemographic characteristics of the study sample are given in Table 1. The mean age was 49.8 years (*SD* = 17.37; range = 18–94), and 55.7% of the sample were women.

### Normative Values

Normative scores for the DS-I total score are given in Table 2, based on the total sample. It shows what percentage of the population reaches the indicated value or lower on the DS-I. 13.5% of the general population reported demoralization scores that were one standard deviation above the mean value in our sample. Because the threshold (29.46) defined in this way is close to the commonly used cut-off score (≥30) (Kissane et al., 2004) both approaches yield similar results.

**Table 2:** Normative raw values of the DS-I total score stratified by age and sex.

	**Total**	**Men**						**Women**					
**DS-I**	**18–94 yr**	** <30 yr**	**30–39 yr**	**40–49 yr**	**50–59 yr**	**60–69 yr**	**≥70 yr**	** <30 yr**	**30–39 yr**	**40–49 yr**	**50–59 yr**	**60–69 yr**	**≥70 yr**
**Percentile**	***N* = 2,407**	***N* = 174**	***N* = 163**	***N* = 182**	***N* = 211**	***N* = 182**	***N* = 154**	***N* = 209**	***N* = 213**	***N* = 238**	***N* = 249**	***N* = 217**	***N* = 215**
50%	13	13	12	12	12	13	13	14	12	13	15	13	14
75%	21	20	18	19	19	20	19	23	20	21	26	21	25
90%	34	35	34	36	30	30	30	34	35	30	39	31	36
95%	44	42	43	43	39	46	36	50	44	44	50	39	43

*DS-I, Demoralization Scale (I)*.

### Age and Sex Differences

The mean scores and standard deviations and Cronbach's α of the DS-I subscales and total score are presented in Table 3, separated by sex and age groups. *F*-scores and effect sizes are shown in Table 4.

**Table 3:** Mean scores and standard deviations of the DS-I separated by sex and age groups.

		**Total score**		**LoM**		**DHM**		**DYS**		**SoF**	
		*****α***** **=** **0.94**		*****α***** **=** **0.91**		*****α***** **=** **0.89**		*****α***** **=** **0.78**		*****α***** **=** **0.78**	
		***M***	**(*SD*)**	***M***	**(*SD*)**	***M***	**(*SD*)**	***M***	**(*SD*)**	***M***	**(*SD*)**
**Men**											
<30 years	*n* = 174	16.44	(12.77)	2.72	(4.53)	2.83	(3.75)	4.15	(3.61)	6.74	(3.48)
30–39 years	*n* = 163	15.63	(12.58)	2.53	(4.28)	2.77	(3.80)	3.92	(3.04)	6.40	(3.44)
40–49 years	*n* = 182	15.75	(12.39)	2.49	(4.26)	2.81	(3.75)	4.42	(3.05)	6.03	(3.24)
50–59 years	*n* = 211	15.19	(12.21)	2.38	(4.35)	2.79	(3.65)	3.99	(2.80)	6.03	(3.54)
60–69 years	*n* = 182	16.42	(11.40)	2.60	(4.19)	2.84	(3.57)	4.33	(2.99)	6.65	(3.34)
≥70 years	*n* = 154	15.58	(10.58)	2.55	(3.86)	2.80	(3.31)	4.04	(2.78)	6.20	(3.40)
All	*n* = 1,066	15.82	(12.02)	2.54	(4.25)	2.80	3.64)	4.14	(3.05)	6.33	(3.41)
**Women**											
<30 years	*n* = 209	17.91	(13.67)	2.99	(4.50)	3.34	(4.42)	4.96	(3.53)	6.63	(3.62)
30–39 years	*n* = 213	16.18	(13.76)	2.75	(5.02)	3.03	(4.14)	4.80	(3.50)	5.61	(3.37)
40–49 years	*n* = 238	16.79	(12.78)	2.75	(4.42)	3.31	(4.20)	4.75	(3.20)	5.98	(3.43)
50–59 years	*n* = 249	18.79	(14.80)	3.44	(5.24)	3.97	(4.86)	5.02	(3.51)	6.36	(3.38)
60–69 years	*n* = 217	16.47	(11.11)	2.47	(3.86)	3.36	3.61)	4.75	(2.89)	5.89	(3.08)
≥70 years	*n* = 215	18.36	(12.82)	3.40	(4.76)	3.87	(4.31)	4.53	(2.96)	6.56	(3.30)
All	*n* = 1,341	17.44	(13.25)	2.97	(4.67)	3.49	(4.29)	4.80	(3.28)	6.17	(3.38)
**Total sample**											
<30 years	*n* = 383	17.24	(13.27)	2.87	(4.51)	3.11	(4.13)	4.59	(3.58)	6.68	(3.55)
30–39 years	*n* = 376	15.94	(13.25)	2.65	(4.71)	2.91	(3.99)	4.42	(3.33)	5.95	(3.42)
40–49 years	*n* = 420	16.34	(12.61)	2.64	(4.35)	3.09	(4.01)	4.61	(3.14)	6.00	(3.35)
50–59 years	*n* = 460	17.14	(13.77)	2.95	(4.88)	3.43	(4.38)	4.55	(3.24)	6.21	(3.45)
60–69 years	*n* = 399	16.45	(11.23)	2.53	(4.01)	3.12	(3.60)	4.56	(2.94)	6.24	(3.22)
≥70 years	*n* = 369	17.20	(12.00)	3.04	(4.42)	3.43	(3.95)	4.32	(2.90)	6.41	(3.34)
All	*n* = 2,407	16.72	(12.74)	2.78	(4.49)	3.19	(4.03)	4.51	(3.20)	6.24	(3.40)

*M, mean of Demoralization (sub-) scale; SD, standard deviation; α, Cronbach's alpha; DS-I, Demoralization Scale (I); LoM, Loss of Meaning; DHM, Disheartenment; DYS, Dysphoria; SoF, Sense of Failure*.

**Table 4:** *F*-scores and effect sizes of group differences regarding sociodemographic variables on DS-I total score and all subscales.

		**Total score**	**LoM**	**DHM**	**DYS**	**SoF**
Age group	*F*	0.59(*n*.*s*.)	0.62(*n*.*s*.)	0.83(*n*.*s*.)	0.54(*n*.*s*.)	2.19(*n*.*s*.)
	*d*	0.10	0.11	0.13	0.09	0.22
sex	*F*	9.11^**^	5.14^**^	16.76^**^	25.17^**^	1.53(*n*.*s*.)
	*d*	0.12	0.09	0.17	0.21	0.22
Occupation	*F*	50.66^**^	45.90^**^	39.06^**^	27.61^**^	29.27^**^
	*d*	1.04	1.01	0.92	0.79	0.78
Partnership	*F*	63.16^**^	71.38^**^	58.34^**^	18.39^**^	30.12^**^
	*d*	0.33	0.35^*^	0.32	0.19	0.22
Income	*F*	18.03^**^	12.31^**^	12.61^**^	11.74^**^	14.81^**^
	*d*	0.39	0.32	0.36	0.37	0.27
Education	*F*	12.40^**^	11.20^**^	7.52^*^	8.68^**^	14.75^**^
	*d*	0.22	0.21	0.18	0.15	0.25

*DS-I, Demoralization Scale (I); LoM, Loss of Meaning; DHM, Disheartenment; DYS, Dysphoria; SoF, Sense of Failure; For the ANOVAs testing for group differences regarding occupation, partnership, income and education sex and age group were included as covariables*.

* *p = 0.001;*

** *p < 0.001; n.s.: p > 0.05*.

The level of demoralization was significantly higher in women than in men for the total score of the DS-I (*F*_(1,2395)_ = 9.11, *p* = 0.003, *d* = 0.12) as well as the subscales Loss of Meaning (*F*_(1,2395)_ = 5.14, *p* = 0.023, *d* = 0.09), Disheartenment (*F*_(1,2395)_ = 16.76, *p* < 0.001, *d* = 0.17), and Dysphoria (*F*_(1,2395)_ = 25.17, *p* < 0.001, *d* = 0.21). We found no sex difference with regard to the Sense of Failure subscale (*F*_(1,2395)_ = 1.53, *p* = 0.22).

We also found no statistically significant differences in the levels of demoralization between the age groups (see Table 4).

### Group Differences in Sociodemographic Characteristics

We found group differences for all sociodemographic characteristics including occupation, partnership, income, and education (see Table 4). For these calculations age group and sex were controlled. The largest differences in DS-I-scores were found between the different groups of occupational status, with the unemployed individuals having the highest scores on both DS-I total score as well as on all subscales (see Table 5). With regard to partnership, demoralization scores were significantly higher in participants living without a partner on all DS-I scales. Lower income and lower education were also associated with higher scores on DS-I total score and all subscales. Our data further suggest a non-linear trend with the highest educated group having slightly higher scores than the intermediate group on all DS-I scales except Sense of Failure.

**Table 5:** Group differences in sociodemographic variables regarding demoralization scores.

	**Total score**		**LoM**		**DHM**		**DYS**		**SoF**	
	***M***	**(*SD*)**	***M***	**(*SD*)**	***M***	**(*SD*)**	***M***	**(*SD*)**	***M***	**(*SD*)**
**Occupation**										
Full or part time	14.94	(11.25)	2.18	(3.80)	2.65	(3.56)	4.21	(3.04)	5.89	(3.29)
Unemployed	28.17	(18.47)	6.72	(7.00)	6.37	(5.60)	6.53	(4.05)	8.55	(4.00)
Retired	17.25	(12.39)	2.94	(4.53)	3.42	(4.06)	4.44	(2.94)	6.45	(3.39)
Other	18.02	(13.47)	3.22	(4.75)	3.52	(4.29)	5.03	(3.53)	6.25	(3.16)
**Partnership**										
Without partner	19.11	(14.25)	3.67	(5.17)	3.92	(4.54)	4.86	(3.35)	6.66	(3.47)
With partner	14.88	(11.10)	2.10	(3.76)	2.62	(3.48)	4.24	(3.04)	5.92	(3.30)
**Income**										
<1,000	18.92	(14.37)	3.39	(5.14)	3.80	(4.54)	5.05	(3.37)	6.68	(3.54)
1,000–2,000	16.34	(12.32)	2.70	(4.35)	3.09	(3.92)	4.41	(3.18)	6.15	(3.30)
>2,000	13.95	(9.96)	1.95	(3.41)	2.37	(3.09)	3.87	(2.76)	5.76	(3.40)
**Education**										
<10 years	18.31	(13.62)	3.29	(5.00)	3.60	(4.21)	4.71	(3.20)	6.71	(3.56)
10–11 years	15.50	(11.95)	2.33	(4.10)	2.86	(3.87)	4.23	(3.12)	6.08	(3.29)
>11 years	16.53	(12.51)	2.82	(4.28)	3.15	(3.98)	4.71	(3.29)	5.85	(3.27)

*M, mean of Demoralization (sub-) scale; SD, standard deviation; calculations were controlled for age group and sex; LoM, Loss of Meaning; DHM, Disheartenment; DYS, Dysphoria; SoF, Sense of Failure*.

### Correlations Among the DS-I Subscales and Associations With the FACIT-Fatigue and ET Scales

Table 6 shows the correlations of DS-I subscales and total score with FACIT-Fatigue and Emotion Thermometers (ET). The correlations of the DS-I subscale Disheartenment with FACIT and ETs, except ET-anger, are noticeably stronger than for other subscales of the DS-I. It is comparable in magnitude to the DS-I total score. The DS-I-Sense of Failure subscale is relatively weakly associated with FACIT and ET, especially with the distress component of the latter instrument.

**Table 6:** Correlations between DS-I, FACIT-fatigue and ET subscales, as well as intercorrelations of DS-I (sub-) scales.

	**Total score**	**LoM**	**DHM**	**DYS**	**SoF**
FACIT	−0.68	−0.63	−0.66	−0.54	−0.44
ET1 (Distress)	0.37	0.32	0.39	0.37	0.14
ET2 (Anxiety)	0.59	0.54	0.59	0.50	0.32
ET3 (Depression)	0.69	0.64	0.69	0.57	0.38
ET4 (Anger)	0.48	0.40	0.44	0.53	0.23
ET5 (need help)	0.60	0.57	0.60	0.47	0.33
DS-I Total score	-	0.94	0.92	0.80	0.67
DS-I LoM		-	0.87	0.68	0.51
DS-I DHM			-	0.71	0.46
DS-I DYS				-	0.31

*DS-I, Demoralization Scale (I); FACIT-fatigue, Functional Assessment of Chronic Illness Therapy Fatigue Scale; ET, Emotion Thermometers; LoM, Loss of Meaning; DHM, Disheartenment; DYS, Dysphoria; SoF, Sense of Failure; All correlations were significant at the 0.001 level*.

The intercorrelations (also shown in Table 6) of the subscales are relatively strong, with the Sense of Failure subscale again being the least associated with the other subscales.

## Discussion

In our study, we administered the German version of the DS-I to a representative general population sample. The first aim of the study was to provide population-based sex- and age-specific normative values for the DS-I. To our knowledge, to date there are no studies available of the DS-I assessed in representative general population samples. Now, with the normative data derived from a German general population sample, we provide results of a nationally representative sample of 2,407 participants. These normative data can now be used as reference for the interpretation of empirical clinical data.

The internal consistency of the DS-I with Cronbachs Alpha of 0.94 found in our study is excellent and comparable to other studies with clinical groups (Kissane et al., 2004; Mehnert et al., 2011). Striking is the very high correlation between the DS-I total score with the two subscales Loss of Meaning and Disheartenment.

As was to be expected, the participants of this general population study were markedly less demoralized than clinical samples, for example, patients suffering from cancer (Mehnert et al., 2011; Quintero Garzón et al., 2018; Philipp et al., 2020), Parkinson disease (Koo et al., 2018), or patients with suicidal behavior (Costanza et al., 2020).

The second goal of this study was to analyze associations between demoralization and sociodemographic characteristics, fatigue as well as distress, depression, anxiety, anger, and need for support in the general population.

In our population-based sample, women reported higher levels of demoralization than men, which is consistent with previous findings in the literature (Robinson et al., 2015, 2016). This sex difference was found for the total demoralization score as well as for the Loss of Meaning, Disheartenment, and Dysphoria subscales. For the subscale Sense of Failure no sex-based difference could be found.

Age was not associated with demoralization in our sample. There was no linear age trend. For those in the highest age-group, with an age of 70 or above, however, the aforementioned small sex-difference increased substantially with the older women being more demoralized. In contrast, in clinical samples severe demoralization has been reported to be associated with younger age in patients with cancer or a progressive disease (Robinson et al., 2015). An explanation for this inconsistency could be the higher likelihood for severe physical symptoms or limiting health conditions at a higher age in the general population, which have been linked to existential distress (Oechsle et al., 2014).

Occupational status was shown to be associated with demoralization. Group differences between different occupational statuses were significant for all subscales of the DS-I with the unemployed being the most demoralized. This finding is consistent with findings of Lee et al. (2012), who described a significant difference in magnitude of demoralization depending on occupational status. Work has repeatedly been associated with finding meaning in life either through creative expression (Frankl, 1992) or as a source of achievement (Emmons, 2003) and has been named as the second most important source for meaning in life in university students (Fegg et al., 2008).

As it has been repeatedly reported in previous studies (Katz et al., 2001; Mehnert et al., 2011), those who live without a partner describe higher levels of demoralization than those who live with a partner. Family, friends and partners are among the most often mentioned areas relevant to the meaning of life (Fegg et al., 2008). Higher income was associated with lower demoralization which is in accordance with findings describing monthly income as protective factor of demoralization (Lee et al., 2012; Li et al., 2017). This could be due to existential concerns as a result of financial insecurity. Low academic levels predicted stronger demoralization symptoms suggesting a buffering effect that might occur as a concomitant effect of higher education. This is in line with a study of Katz et al. (2001), where lower levels of education were associated with higher scores of demoralization, although relatively small group differences and a non-linear trend make it difficult to reasonably explain this finding.

The link between psychological distress and demoralization was slightly weaker than it has been described in a sample of cancer patients (Mehnert et al., 2011). This may be due to a different interpretation of the term “distress” in the general population compared to medically ill patients. Healthy individuals may consider their everyday situations and daily hassles when answering the distress question, while cancer patients may refer mainly to their disease-related burdens (Hinz et al., 2018). The association of demoralization with depression was comparable to what was found in cancer patients (Mehnert et al., 2011), probably due to the symptomatic overlap of those two constructs. Anxiety was also strongly associated with demoralization and consistent with findings in previous studies (Katz et al., 2001; Mehnert et al., 2011).

### Limitations

The response rate of this study (51.9%) was sufficient but not optimal. Despite the representativeness of the sample in terms of age and sex, we cannot provide information on levels of demoralization in non-responders, which could lead to an unknown bias in our sample. Since it is a sample of the German general population the results are not necessarily generalizable to other countries. No calculation of the construct validity of the DS-I was made using the data from this study. Another limiting factor could be the lack of clinical data of the present sample.

### Conclusions

The normative values provided by this study can be used in clinical routine and research. Associations between demoralization and sociodemographic and psychological variables are mostly consistent with the findings in cancer patients. Future research should evaluate specific differences regarding demoralization between the general population and clinical populations.

## Data Availability Statement

The data analyzed in this study is subject to the following licenses/restrictions: The data are not publicly available due to privacy or ethical restrictions. Requests to access these datasets should be directed to leonhard.quintero@medizin.uni-leipzig.de.

## Ethics Statement

The studies involving human participants were reviewed and approved by the ethics committee of the University of Leipzig approved the study. The patients/participants provided their written informed consent to participate in this study.

## Author Contributions

LQ, AH, and AM-T contributed to conception and design. LQ and AH contributed to data analysis and participated in the creation of tables. LQ wrote the majority of the manuscript. All authors contributed to manuscript revision, read, and approved the submitted version. All authors were involved in the writing process and reviewed the manuscript.

## Conflict of Interest

The authors declare that the research was conducted in the absence of any commercial or financial relationships that could be construed as a potential conflict of interest.
